# Salinity tolerance of Round Goby: Informing invasion potential in North American coastal watersheds

**DOI:** 10.1371/journal.pone.0316327

**Published:** 2025-04-25

**Authors:** Kelsey Alvarez del Castillo, Suresh A. Sethi, Eugene Won, John Maniscalco, Richard Pendleton, Eliza Ryan, Lars G. Rudstam

**Affiliations:** 1 Department of Natural Resources and the Environment, Cornell University, Ithaca, New York, United States of America; 2 Aquatic Research & Environmental Assessment Center, Brooklyn College, Brooklyn, New York, United States of America; 3 Department of Animal Science, Cornell University, Ithaca, New York, United States of America; 4 Division of Marine Resources, New York State Department of Environmental Conservation, Kings Park, New York, United States of America; 5 Department of Natural Resources and the Environment, Cornell University, Ithaca, New York, United States of America in cooperation with the Division of Marine Resources, New York State Department of Environmental Conservation, New Paltz, New York, United States of America; 6 Cornell University, The Office of Undergraduate Biology, Ithaca, New York, United States of America; Universidade de Brasilia, BRAZIL

## Abstract

Since being introduced into the Laurentian Great Lakes in the 1990s, round goby (*Neogobius melanostomus*) has spread rapidly, reaching the Hudson River Estuary, NY in 2021. To address the expansion potential into saline environments from this North American coastal invasion front, we experimentally assessed the salinity tolerance of adult round gobies. Water temperatures vary widely in temperate aquatic ecosystems, and our study is the first to investigate the effect of temperature on the salinity tolerance of round goby, conducting tolerance trials at three temperatures: a preferred temperature of 20°C, 26°C reflective of summer conditions, and 5°C reflective of winter conditions. Adult round gobies were subjected to weekly salinity increases of 3 parts per thousand (ppt), concluding at 33ppt. Study specimens were monitored for stress cues (behavior changes and color changes), and mortality. We found significant salinity tolerance differences dependent on water temperature, with the highest tolerance at 5°C and the lowest tolerance at 26°C. By 30ppt, survival was 87% at 5°C and only 7% at 26°C. Based on mortality results, round goby expansion may occur year-round into brackish portions (<21ppt) of the Hudson River Estuary as far south as the Harlem River. This would open access to portions of Long Island Sound, potentially rendering other coastal watersheds to be at risk of invasion. However, temperature-dependent salinity tolerance findings suggest round goby expansion potential into high salinity habitats may be seasonally dependent, with expansion opportunities occurring in colder months and expansion barriers occurring in warmer months. To assess longer term survival and body condition, another experiment maintained round gobies at four sustained salinities (≤ 21ppt) for ten weeks at 20°C. Growth and hepatosomatic index at 21ppt were significantly lower (p<0.001) than at 1, 9, and 15ppt, indicating sustained exposure to higher salinities may affect energy stores, potentially limiting establishment potential.

## Introduction

Round goby (*Neogobius melanostomus)* is one of the fastest spreading fish species in North America and considered one of the ten invasive species with the highest ecological impact within the Great Lakes region [1]. Native to the Ponto-Caspian region, round goby were first introduced via ship ballast water into the St. Clair River, Michigan, in 1990 [2] and colonized all five of the Laurentian Great Lakes in less than ten years [3,4]. Round goby quickly expanded outward into major riverine systems such as the Des Plaines River, Illinois to the West, where they have continued southward expansion into the Mississippi River [5]. The rapid expansion of round goby has recently extended eastward into freshwater portions of ecologically and economically important coastal estuarine systems including the St. Lawrence and Hudson Rivers [4,6]. As a brackish water tolerant species in their natal range, round goby may have the capacity to tolerate salinities within coastal estuaries and connected marine waters in North America. Thus, continued expansion and establishment of round goby into coastal watersheds has elevated concerns over potential impacts to these ecosystems and the potential for further spread as these systems serve as pathways to other rivers and watersheds.

A suite of ecological consequences has been documented in North American waterways where round goby has proliferated. Round goby consume a variety of benthic invertebrates causing declines in macroinvertebrate assemblages [7–9] and can outcompete small, native benthic fish species leading to a decline in fish diversity [10–12]. Further, round goby consume eggs of ecologically and economically significant fish species such as walleye (*Sander vitreus*) [13], lake trout (*Salvelinus namaycush*) [14], and smallmouth bass (*Micropterus dolomieu*) [15], and have been linked as vectors to diseases such as viral hemorrhagic septicemia [16–18] and avian botulism [19,20]. Round goby consume invasive dreissenid mussels, leading to alterations in water quality and the transfer of heavy metals and polychlorinated biphenyls to higher trophic levels [21–23]. However, this consumption creates new trophic linkages, increasing availability of energy stored in mussels to higher trophic levels [24,25]. Indeed, round goby has become a prominent food source for predatory fish species including lake trout, yellow perch (*Perca flavescens*), and smallmouth bass, leading to increased growth in these species [8,15,26–28]. Diet shifts towards high reliance on round goby have also been documented in non-fish species such as double-crested cormorants (*Phalacrocorax auritas)* and Lake Erie watersnake (*Nerodia sipdeon insularum)* [29,30].

**Fig 1:**
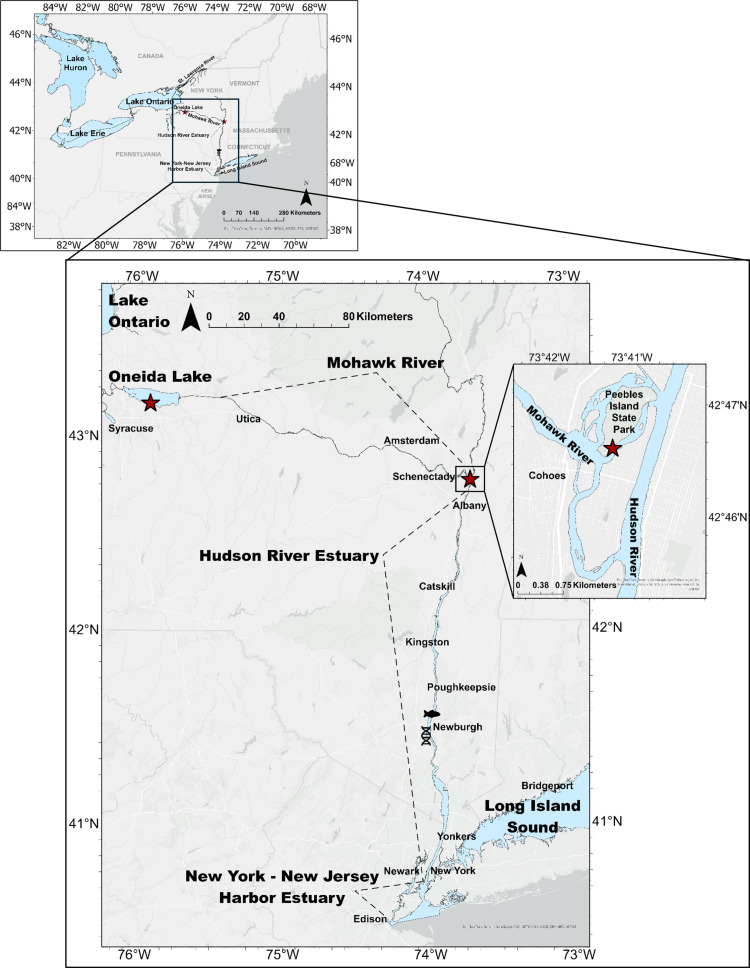
Connected waterbodies of New York State along the round goby invasion front. Sampling locations at Oneida Lake and the confluence of the Mohawk and Hudson River are depicted with red stars. The farthest south a round goby has physically been captured in the Hudson River Estuary is denoted by a fish icon whereas the farthest south the New York State Department of Environmental Conservation has had a positive round goby eDNA detection is denoted by a helix icon. This map was created using ArcGIS Pro version 3.1.0 from esri (https://www.esri.com/en-us/home). All map layers are under public domain. The basemap layer (Light Gray Reference) was accessed from the ArcGIS Pro basemap gallery. The GIS map layer containing hydrography was compiled from NYS GIS Clearinghouse – NYS Hydrography Layer (https://data.gis.ny.gov/maps/sharegisny::nys-hydrography/about) and from the U.S. National Ice Center – Great Lakes Data Support files (https://usicecenter.gov/Products/GreatLakesData). Credits: ESRI, TomTom, Garmin, SafeGraph, GeoTechnologies, Inc, METI/NASA, FAO, NOAA, USGS, EPA, NPS, US Census Bureau, USDA, USFWS.

The extent to which round goby can tolerate salinities in North American coastal watersheds and spread downstream into estuary and marine environments is currently unknown. Presently, round gobies are known to occupy only two North American coastal estuaries: the St. Lawrence Estuary, Canada and the Hudson River Estuary, NY. The St. Lawrence River spans from Eastern Lake Ontario to the Gulf of St. Lawrence with the estuarine portion beginning at Quebec City [31]. To date, the farthest downstream round gobies have consistently been documented in the St. Lawrence Estuary is downstream of Ile d’Orléans, with salinity levels ranging from 2–5 ppt. Sporadic catches have occurred 60 km downstream of this location, up to 10–12 ppt (O. Morissette, Assistant Professor-Université du Québec à Chicoutimi, personal communication). Further south, the Hudson River Estuary spans from the Federal Dam at Troy, New York, downstream 246 km to the Battery, where the estuary meets New York Harbor, NY. The salt front fluctuates between Poughkeepsie and Newburgh, approximately 100 river kilometers from the ocean, depending on tidal cycles and precipitation [32], but in seasons of high freshwater inflow it can be as far south as Tappen Zee, only ~37km from the New York Harbor and connected marine waters. Saltwater influence is prominent south of Newburgh (river km 98). Since the first documented catch in the Hudson River Estuary in summer 2021 [6], round gobies have been caught just north of Newburgh (river km 105) and positive eDNA detections have occurred just south of Newburgh (river km 92) at salinities < 0.15 ppt (Fig 1, R. Pendleton, New York State Department of Environmental Conservation, personal communications).

While current monitoring programs have not detected round gobies in higher salinity waters of coastal North American watersheds, the ability to adapt to increasing salinities is a concern given the salinity levels of their natal and Eurasian invaded range. The native range encompasses the Black Sea averaging 18–22 ppt depending on depth [33], the Sea of Azov averaging 12 ppt [34], and the Caspian Sea averaging 13 ppt [35]. Furthermore, the salinity tolerance of populations may be increasing as round gobies are expanding and adapting to higher salinities throughout the invaded Baltic Sea region [36,37], reflected by documented catches of round gobies in 30 ppt in the Gothenburg Harbor (west coast of Sweden) [38].

The salinity tolerance of fishes is linked to energy demands for osmoregulation, with temperature influencing this physiological process. Osmoregulation is energetically costly, utilizing approximately 10 to >50% of the resting metabolic rate [39,40]. Lower energy expenditure ensues when the internal environment of the fish is iso-osmotic to that of the external environment, which occurs at 9–12 ppt for most species [39,41]. Indeed, the fastest growth rate of round goby has been documented at 5–10 ppt in laboratory conditions [42,43], and round gobies are typically larger in brackish waters compared to those in freshwater [44,45]. Temperature influences physiological processes such as osmoregulation, through shifts in metabolic rates and is likely an important driver of round goby salinity tolerance [46,47]. Round gobies are not only euryhaline, but also eurythermal, surviving in temperatures ranging from -1–30°C [48]. Lee & Johnson [49] and Skazkina & Kostyuchenko [50] found that at warmer temperatures (20–24°C) metabolic rates of round goby are 5–6 times higher than at cooler temperatures (0–3.5°C). A temperature preference and avoidance study conducted by Christensen et al. [51] reported preferred temperatures of round goby occurred between 20–22°C, with temperature avoidance occurring at 24°C. Studies examining maximum feeding rates as a proxy for preferred temperature found peak consumption rates ensued at 18–26°C, dependent on acclimation temperatures. Round goby experience a drastic bioenergetic decline past 24–26°C due to reductions in maximum food consumption rate and increases in standard metabolic rate [49,52]. Given the wide thermal preference and tolerance of round goby; assessing the impact of water temperatures on salinity tolerance is important for understanding expansion risk in invaded estuarine ecosystems.

Several laboratory experiments have investigated the salinity tolerance of round goby in natal and invaded systems. However, these studies have yielded variable results of round goby salinity tolerances that may be dependent on the origin of specimens, acclimation methods, and sample sizes. Laboratory studies that used round gobies taken from brackish waters in their invaded Eurasian range, primarily from the Baltic Sea, found higher salinity tolerances than those using round gobies sourced from freshwater systems. Round gobies collected from the western Baltic Sea at 10 ppt began showing significant signs of osmotic stress at 25 ppt with over 50% survival at 30 ppt [53,54]. Puntila-Dodd et al. [55] also utilized round gobies from the western Baltic Sea (11 ppt), and after acclimation at 30 ppt for over four weeks, reported 100% survival. Similarly, Hempel & Thiel [43] experienced 100% survival at 7.5, 15, and 30 ppt after 12 weeks with round gobies collected at 3.2 ppt along the Kiel Canal, near Rendsburg, Germany. In contrast, laboratory studies sourcing fish from the invaded North American Laurentian Great Lakes, which have not faced selection pressure for marine environments, have yielded lower salinity tolerance results. Karsiotis et al. [42] sourced round gobies from the Great Lakes proper (freshwater) and found 0% survival at 30 ppt and only 10–30% survival at 25 ppt in stepwise salinity increase experiments, increasing tanks 5 ppt every 3 days, over a three week period. Ellis & Macisaac [56] also found 0% survival of Great Lakes round gobies at 30 ppt after 48 hours, possibly the results of transferring specimens from freshwater directly to 30 ppt without acclimation. The variable results from these existing laboratory trials indicate that questions remain about salinity tolerances of this species, especially under conditions more reflective of in situ habitats in temperate coastal estuaries in North America.

Here, we examine the salinity tolerance of round gobies sourced from the coastal invasion front in North America across ranges of salinities using laboratory-controlled trials. To further expand upon previous salinity tolerance trials, we are the first to examine the effect of water temperature on salinity tolerance of adult round gobies. With water temperatures varying widely throughout temperate aquatic ecosystems, we provide novel insight into the potential for seasonal dependent expansion in estuarine systems. Adult round gobies collected from populations in the vicinity of the Hudson River Estuary were subjected to regular salinity increases of 3 ppt per week, the most gradual transition in any round goby salinity trial to date, and concluded at 33 ppt after 12 weeks. We repeated trials at three temperature regimes reflective of the Hudson River Estuary: preferred temperature of 20°C (henceforth ‘preferred’), 26°C reflective of summer conditions (henceforth ‘warm-water’), and 5°C reflective of winter conditions (henceforth ‘cold-water’). The preferred temperature of 20°C was selected based off of the temperature preference and avoidance study conducted by Christensen et al. [53]. The primary focus of the study was to understand the capacity of round goby to tolerate salinity levels by monitoring mortalities throughout the trials. To aid in the understanding of fish condition throughout the trials, we also monitored specimens for cues of stress through behavioral changes and coloration changes. To provide further insight into long-term survival and establishment potential, adult round gobies were acclimated to salinities of 1, 9, 15, and 21 ppt and held at those salinities for approximately ten weeks after which change in biomass and hepatosomatic indices were recorded. The results of this study provide insights into the capacity of North American round goby to inhabit brackish and marine waters, informing the expansion potential of this rapidly proliferating and adaptable fish species.

## Methods

We ran a suite of experiments to test the salinity tolerance of round gobies sourced from the eastern edge of the invasion front in New York State. We attempted to collect round gobies directly from the Hudson River Estuary, however, catch rates in the field were too low and an adequate sample size for tolerance trials was not obtained. In lieu of this, we collected round gobies from the confluence of the Mohawk and Hudson Rivers, and from Oneida Lake (Fig 1). Oneida Lake is proximal to the experiment location which allowed for easily accessible and convenient fish capture, minimizing transport stress for study fish. Round gobies in the Hudson River Estuary are suspected to be of Oneida Lake or Mohawk origin, as documented catches show timely eastward expansion directly along the Erie Canal which connects Oneida Lake to the Hudson River [6,57]. It is hypothesized that larval drift played a significant role in downstream dispersal from the Mohawk River to the Hudson River Estuary, but there has been no population genetics work to confirm origin of Hudson River Estuary gobies [[6], R. Pendleton, New York State Department of Environmental Conservation, personal communications]. Thus, our initial experiment examined salinity tolerance of round gobies at a preferred temperature of 20°C, in addition to assessing whether salinity tolerance differed between Oneida Lake and the Mohawk/Hudson River confluence (henceforth ‘Hudson River’) specimens. As we did not detect any differences in response by Oneida Lake and Hudson River fish (see results), we ran the two additional salinity tolerance trials at warm (26°C) and cold (5°C) temperatures with only Oneida Lake round gobies. Finally, we ran a sustained salinity exposure experiment with Oneida Lake round gobies to assess survival, growth, and body condition at constant salinity levels in 20°C waters.

### Fish collection, acclimation, and holding

Collection of round gobies occurred under New York State Department of Environmental Conservation License to Collect #2995 and Cornell University Animal Care Protocol 2015–0021, transport of fish occurred under invasive species transport permit #07-22-003 from New York State Department of Environmental Conservation, and experimentation was conducted under Cornell University Animal Care Protocol 2022–0141 and 2006–0088. Adult round gobies for the initial experiment at 20°C were collected in September 2022 from the Mohawk River near Waterford, NY (42°46’38“N, 73°41’15”W) and from the shoreline of Oneida Lake, NY at Shackelton Point (43°10’26”N, 75°55’50”W) using a backpack electrofishing unit and a beach seine, respectively. Study specimens used in the 26°C, 5°C, and sustained exposure trials were sourced from Shackelton Point in Oneida Lake in June 2023.

Upon capture, fish were acclimated to laboratory conditions in 800 liter circular aerated holding tanks for a minimum of two weeks at 1 ppt (initial experiment 2022) or 6 ppt (all subsequent experiments in 2023) salinity (Instant Ocean, Spectrum Brands Inc, mixed with dechlorinated tap water) at ambient temperature (~20°C) to minimize the risk of fungal infection or disease transmission [58,59]. Subsequently, to reach other experimental temperatures, fish in cold-water holding tanks were subjected to a temperature decline to 5°C over the duration of five days whereas fish in warm-water holding tanks were subjected to a temperature increase to 26°C over the course of two days.

Fish were held in 151-liter volume aquaria filled with dechlorinated tap water for the salinity tolerance experiments at 20°C, 26°C, and 5°C water temperatures. For the sustained exposure experiment, 38-liter volume aquaria were used. Water temperatures in the 20°C and 5°C experiments were maintained via ambient temperature in climate-controlled rooms. HiTauing 25W small aquarium heaters were used in the 26°C experiments. All tanks were set up with PVC shelters, sponge filters with aeration, and a mix of sanitized gravel and sand substrate was placed on the bottom of the tanks and seeded with nitrifying bacteria (API® Quick Start) prior to the start of the experiment. PVC shelters consisted of halved two-inch PVC that were approximately four inches in length. A plastic mesh divider was placed in all 151-liter tanks, providing two tank-level compartments for these experiments; 38-liter tanks did not use dividers. Cardboard surrounded three out of the four sides of all tanks to reduce light penetration and fish stress, in addition all tanks had corrugated plastic lids to minimize evaporation and fish escapes [S1 File]. Fish were held with photoperiods of 12–14 hours.

### Salinity tolerance trials

#### Preferred temperature (20°C); Oneida Lake versus Hudson River fish.

Fish [total length mean=60mm; range=36–107mm, sd=12] were randomly distributed into experimental and control aquaria with 10 fish from Oneida Lake on one side of the plastic mesh divider and 10 fish from the Hudson River on the other side, totaling 20 fish per aquaria. Ten experimental tanks (n=200 individuals) and three control tanks (n=60 individuals) were used. Experimental tanks began at 0 ppt and were increased by 3 ppt every week until 33 ppt was reached, totaling 12 weeks. Control tanks were maintained at 1 ppt to minimize the risk of fungal infection or disease transmission [58,59]. Water temperature, pH, specific conductance (mS/cm), and dissolved oxygen (% and mg/L) were checked daily using a Hydrolab®DS5 (OTT HydroMett, USA). Ammonia and nitrate levels were checked daily using API® test kits. To maintain water quality a 50% water change was conducted weekly in conjunction with the increase in salinity. Fish were fed 2% of their body mass, six days a week, with 2 mm pellets (#2 sinking starter crumble for sturgeon, Skretting USA, Tooele, UT). As appetite was observed to decline as the salinity trials progressed, experimental fish were fed *ad libitum*, six days a week. Aquarium were held at the target temperature of 20°C with minor variation in daily temperatures (mean=20.6°C; range=19.16-21.97°C). One control tank was excluded from the results due to an isolated, lethal fungal infection in week eight of the experiment.

#### Warm (26°C) and cold (5°C) temperature regimes.

Following our initial salinity tolerance experiment at a preferred water temperature of 20°C in 2022, we repeated the experiment at 26°C and 5°C, run in parallel in 2023. Finding no difference in survival between Oneida Lake and Hudson River round gobies in our initial experiment (see results), all subsequent fish were sourced only from Oneida Lake. Study specimens (total length mean=57mm; range=42–87mm, sd=7) were randomly distributed into experimental and control aquaria with 10 fish on either side of the plastic mesh divider, totaling 20 fish per 151-liter aquarium. Five experimental tanks (n=100 individuals) and three control tanks (n=60 individuals) were used for each of the two temperature treatments. Experimental tanks started at 6 ppt for 24 hours then increased to 9 ppt for one week. Experimental tanks started at 6ppt to shorten the duration of the trial; experimental fish were behaving normally, and no mortality occurred at this salinity (see results) in the initial trial at 20°C. Thereafter, salinity levels in experimental tanks continued to be increased 3 ppt every week until 33 ppt was reached, totaling 9 weeks. Control tanks were held at 1 ppt. Daily variations in water temperatures were small for both 5°C (mean=5.22°C; range=4.71–6.90°C) and 26°C (mean=26.40°C; range=24.65–26.90°C) treatments. Water quality maintenance protocols followed those described above in the initial experiment with a preferred water temperature of 20°C. Fish in 26°C tanks were fed six days a week at 2.5% of their body mass, to account for increased metabolic rates in warmer temperatures [49,51] and as appetite declined throughout the trial, specimens were fed *ad libitum*. Fish in 5°C tanks were fed *ad libitum* due to minimal appetite throughout the duration of the trial.

Two cold-water control fish, one in week five and one in week seven, were isolated with signs of an external fungal or bacterial infection and subsequently died. An external parasite, hypothesized to be an encysted digenetic trematode, on round gobies in the cold-water treatment appeared in one experimental tank during week eight and thereafter spread throughout six tanks: five experimental and one control. However, infected individuals were behaving normally, and no clearly infected individuals died throughout the course of the trial.

#### Sustained salinity exposure.

A sustained exposure experiment was conducted in 2023, focusing on round goby survival, growth, and body condition at constant salinity levels for an extended period. Six round gobies collected from Oneida Lake (total length mean=69mm; range=49–90mm, sd=9) were placed in each 38-liter aquarium utilized in this experiment. The target water temperature for this experiment was maintained at 20°C with minimal daily fluctuations (mean=20.41°C; range=18.84–22.51°C). Three tanks were used for each salinity treatment of 1 (control), 9, 15, and 21 ppt (total of 18 fish per treatment). All experimental tanks started at 6 ppt for 24 hours then salinity was increased 3 ppt every two days until tanks were at the assigned endpoint salinities of 9, 15, and 21 ppt. Experimental fish were then held at endpoint salinities for 9–10 weeks until the experiment was terminated. Due to the stepwise salinity increases, endpoint salinities were reached at different days; 9 ppt was reached earliest and maintained for 10 weeks, 15 ppt maintained for 9.5 weeks, and 21 ppt maintained for 9 weeks. Acclimation, holding, daily water quality, and feeding protocols for this experiment followed those of previous experiments.

### Data collection

To assess behavioral and physiological changes associated with fish stress due to increasing salinities, without physically handling and disturbing fish, we implemented a ‘vitality score’ monitoring protocol. Vitality scores are commonly used in commercial fisheries and consist of quick, simple, and non-invasive assessments to measure fish condition to predict release mortality [60]. We created a non-invasive vitality score approach, consisting of three separate categories: coloration, reaction, and appetite. Based on preliminary work, we observed that round goby coloration changed from uniform and light, to dark and mottled when placed in high saline environments. Change in coloration is a known indicator of stress in fish, and other goby species have shown this shift from light to dark coloration in stressful conditions [61–64]. Therefore, coloration was assessed as the proportion of fish with light, uniform coloration. Fish in aquaculture settings undergo learning behaviors such as associating stimuli (i.e., visual or auditory cues) with food, which can serve as an indicator for animal welfare [65–67]. Round gobies quickly became habituated to laboratory conditions and learned when researchers stood near tanks, feeding would occur shortly thereafter. Because of this conditioning, we used ‘reaction’ as a metric which was assessed by observing whether study fish actively reacted to the presence of laboratory staff; a researcher would sit in front of the tank for one minute and count the number of individuals that swam forward in the tank. Lastly, appetite decline is another important indicator of stress and is easy to quantify [68,69]. Appetite was assessed as the proportion of fish that were actively eating during a feeding event. Vitality scores for each category were calculated as the proportion of individuals in each study replicate (a partitioned section of a 151-liter tank) exhibiting healthy characteristics; non-stressed coloration, individuals swimming forward when a person stands in front of the tank, and individuals eating (see S2 File for a visual difference between a healthy versus stressed goby). Vitality scores were calculated on days two, four, and seven at each salinity level, starting at 9 ppt in the preferred temperature trial (20°C) and 6 ppt in the warm (26°C) and cold (5°C) temperature trial. We standardized vitality score measurement efforts by utilizing the same observer, scoring metrics in the same order (color, reaction, appetite), and ensuring lighting and tank conditions were undisturbed during observation periods. Vitality scores were not implemented for the sustained exposure experiment.

All tanks were checked twice daily for mortalities during salinity trials and dead individuals were removed. At the conclusion of the experiments, control fish and any remaining experimental fish were euthanized using a lethal dose (250mg/L) of buffered tricaine methanesulfonate (MS-222; Syndel USA). Study fish were measured for total length (mm) and weight (0.001g) at the start and immediately following mortality or termination of the experiments. In the sustained exposure experiment, we used hepatosomatic index (HSI) as an indicator of energetic condition [70–73]. The HSI is the ratio of liver weight to total wet weight of a specimen; low HSI indicates loss of energy reserves and poorer condition. Therefore, livers of all fish in the sustained exposure experiment that were alive at the end of the experiment were excised and weighed (0.001g) to calculate HSI. Fish that died prematurely were measured for total length and weight, but these specimens were excluded from HSI calculations.

### Data analysis

All statistical analyses were run in RStudio 2023.12.0+369 (Posit Software, PBC). We used log-rank tests to assess for equivalency of Kaplan-Meier survival curves implemented in the *survminer* package [74]. Differences in vitality scores between salinity levels and temperature trials were analyzed using proportion tests. For the sustained exposure experiment, a Kruskal-Wallis test was used to compare pre-experiment weights and post-experiment weights between salinity levels; a Dunn’s test was used for post-hoc analysis in post-experiment weights. Wilcox tests were used to assess changes in biomass at each salinity treatment. Finally, a linear mixed effects regression was used to examine differences in HSI, with salinity as a fixed effect and tank as a random effect using the *nlme* package [75]. Subsequently, Tukey post-hoc comparisons was utilized to identify group differences using the *emmeans* package [76].

## Results

### Preferred temperature (20°C); Oneida Lake versus Hudson River fish

**Fig 2:**
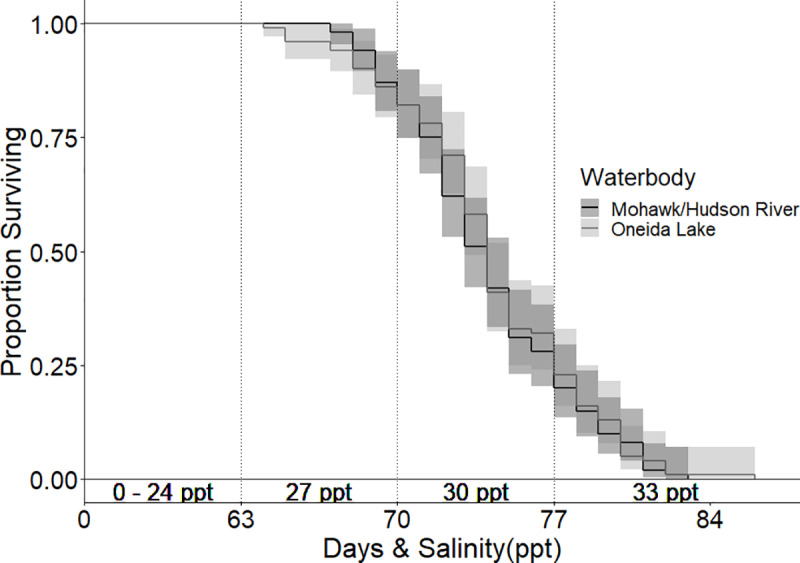
Survival curves for Oneida Lake and Mohawk River round gobies challenged with increasing salinities at 20°C. A log-rank test confirmed there was no significant difference in mortality rates of gobies between waterbodies (p=0.56).

**Fig 3:**
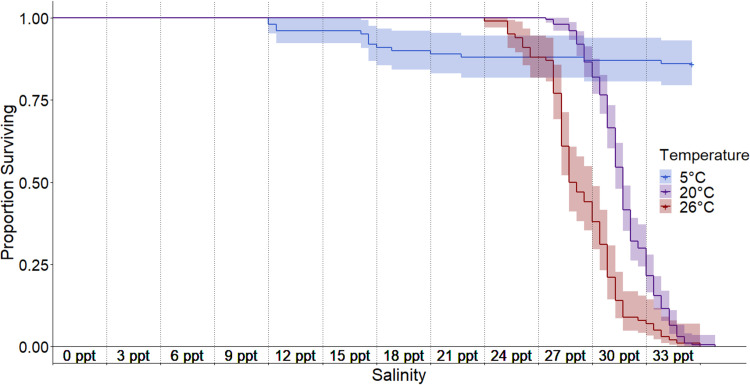
Daily survival curves of experimental round gobies at three temperature treatments. Survival was significantly different (pairwise Log-Rank, p<0.0001) amongst all pairwise comparisons of experimental temperature treatments. There was 100% survival in 20°C and 26°C control groups, resulting in a significant difference between survival in experimental and control groups. There was no significant difference (p=0.26) between experimental (overall 86% survival) and control (overall 92% survival) groups in the 5°C treatment.

**Fig 4:**
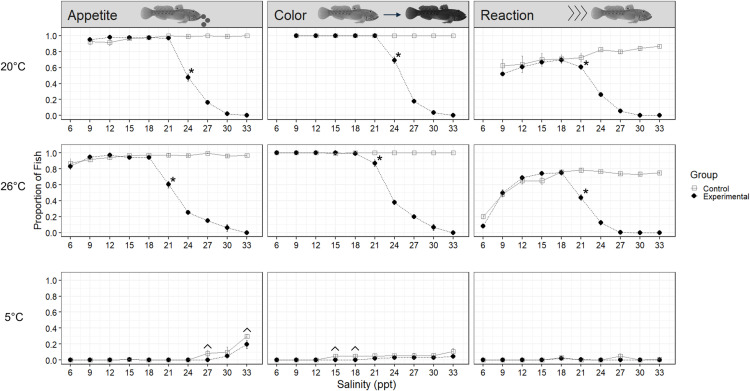
Average proportion of round goby exhibiting non-stressed behavior (eating, light coloration, reacting) across salinity levels at three temperature treatments. A significant, continuous, divergence (proportions test, p<0.02) between control and experimental scores begins at the asterisk (*). A significant difference in experimental and control scores in the cold-water treatment is denoted by ^.

Results from our experiment investigating salinity tolerances of Oneida Lake and Hudson River round goby at a preferred temperature of 20°C [49,51] found no significant difference in survival between the two source populations (log-rank test, *p*-value=0.56; Fig 2). Thus, we combined survival data from the two populations to evaluate salinity tolerance in this experiment. We found 100% survival in 20°C up to salinities of 24 ppt, nine weeks into the experiment (Fig 3). Cumulative survival declined to 82.5% at 27 ppt and to 21.5% at 30 ppt. One individual (0.5%) survived the terminal experiment week at 33 ppt but perished after being held for an additional three days at 33 ppt. One control tank was removed from analysis due to a lethal fungal infection, however, no remaining control fish died throughout the trial. In terms of fish exhibiting signs of stress, vitality scores for experimental fish significantly diverged from control fish scores (Fig 4) beginning at 21 ppt for the ‘reaction’ metric at 21 ppt (χ^2^=5.49, df=1, p=0.019), and at 24 ppt for the ‘appetite’ metric (χ^2^=106.31, df=1, p<0.001) and ‘coloration’ metric (χ^2^=47.83, df=1, p<0.001).

### Warm (26°C) and cold (5°C) temperature regimes

Round goby salinity tolerance was lower in the warm-water treatment and higher in the cold-water treatment, compared to the preferred temperature treatment. A pairwise log-rank test confirmed there was a significant difference in survival rates between all three temperature treatments (p<0.001). In the warm-water (26°C) treatment, we found 100% survival up to 18 ppt (Fig 3). Survival declined to 99% at 21 ppt, 88% at 24 ppt, 38% at 27 ppt, and 7% at 30 ppt. The last individual in the warm-water treatment died on the last day of the experiment (day seven at 33 ppt). Vitality scores for treatment fish significantly diverged from control fish (Fig 4) for all three metrics at 21 ppt (appetite: χ^2^=77.99, df=1, p<0.001; coloration: χ^2^=23.76, df=1, p<0.001; reaction: χ^2^=52.68, df=1, p<0.001). Compared to the preferred temperature experiment, treatment fish showed signs of stress earlier in the warm-water treatment.

In contrast to the preferred (20°C) and warm (26°C) water treatments, the salinity tolerance of round gobies in the cold-water treatment (5°C) was significantly higher (Fig 3). The first experimental fish mortality did occur earlier than in the warm temperature with two deaths at 12 ppt (98% survival); however, few and sporadic mortalities followed with a total of 12 deaths by 21 ppt (88% survival) and ended with 86% survival after a full week at 33 ppt. Background mortality occurred in the cold-water control tanks resulting in 92% survival through the end of the nine week trial, leading to no significant difference in survival between experimental and control fish (log-rank test: *p-*value=0.26). A significant increase in appetite occurred in both experimental fish (χ^2^=17.09, df=1, p<0.001) and control fish (χ^2^=20.46, df=1, p<0.001) throughout the trial, with control fish beginning to eat at week seven and experimental fish at week 8 (Fig 4). Due to high survival through the end of the cold-water salinity trial, we extended the experiment for an additional two weeks using 20 randomly selected experimental and control fish held in separate tanks. No additional mortalities occurred in this extended period for neither control fish (1 ppt) nor experimental fish maintained at 33 ppt.

### Sustained salinity exposure

**Fig 5:**
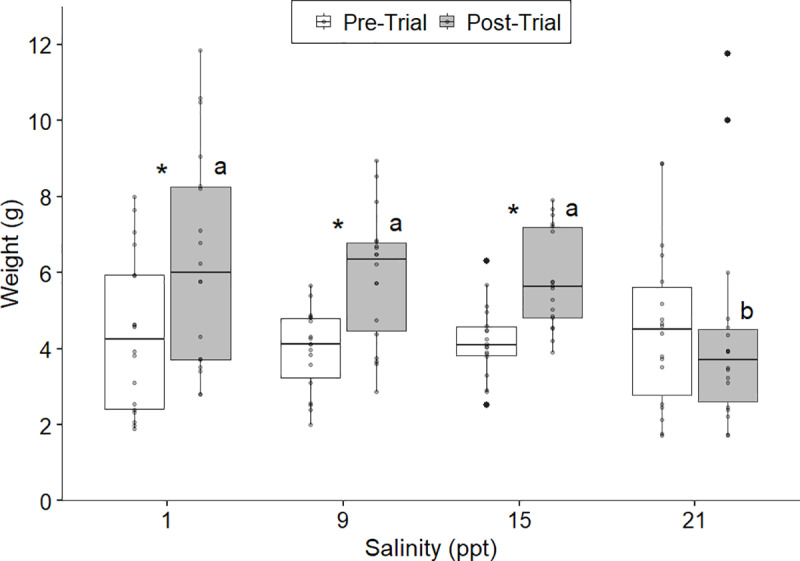
Weight (g) of round goby pre- and post- theexperiment at sustained salinities. Significant differences between pre and post (p<0.05) weights are denoted by an asterisk. Significant differences in post-experiment weights between salinity treatments are designated with different lower-case letters (a versus b: p<0.02); those with identical letters did not differ (p>0.05). At 21 ppt, there was no significant difference between pre and post weights and post-weights were significantly lower than the other three salinity treatments.

**Fig 6:**
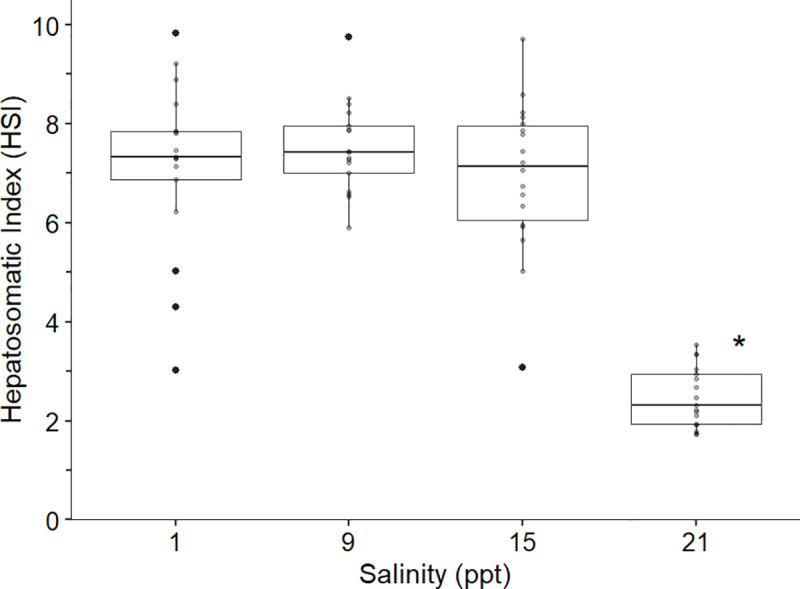
Hepatosomatic index (HSI) of round goby at the termination of the sustained salinity experiment. Significant differences (p<0.001) are denoted by an asterisk.

Finally, the last experiment exposing round gobies to sustained salinity levels resulted in high cumulative survival at all treatments but indicated a threshold salinity effect in terms of growth and HSI. We observed 100% survival of fish held at 9 ppt (one fish escaped a tank and was excluded) and 15 ppt, and 94% survival of fish held at 21 ppt throughout the 10-week trial. A single control specimen died of unknown causes (i.e., no external signs of stress or infection). Fish across the control, 9 ppt, and 15 ppt treatments exhibited significant increases in biomass (Wilcox test: control, 1 ppt: p=0.048, change in median weight = 1.76 g; 9 ppt: p=0.0016, change in median weight = 2.22 g; 15 ppt: p<0.001, change in median weight = 1.54 g) throughout the 10-week trial (Fig 5). However, treatment fish held at 21 ppt did not exhibit any significant change in biomass from the start to end of the trial (p=0.47, change in median weight = -0.80 g), indicating study specimens may have had increased stress levels and reduced energy stores at this salinity level. Additionally, there was no significant difference (p>0.05) in post-experiment weights between 1, 9, and 15 ppt, however post-weights at 21 ppt were significantly lower (p<0.02) compared to the other treatments. Post-mortem analysis of adults showed individuals held at 21 ppt had significantly lower HSI (p<0.001), compared to control tanks (1 ppt) and experimental treatments of 9 and 15 ppt (Fig 6). A Kruskal-Wallis test confirmed there was no significant difference (χ^2^=0.26, df=1, p=0.97) in fish weights at the start of the experiment amongst salinity treatments.

## Discussion

Results from our salinity tolerance experiments for round goby sourced along the eastern New York invasion front provides insight into the expansion potential of this proliferating species into coastal estuarine systems in North America. We found that water temperature significantly affects the salinity tolerance of adult round goby, suggesting spread risk potential may depend on seasonal conditions. Increased mortality began at 24 ppt and 27 ppt for 26°C and 20°C, respectively. However, behavior indices indicate that round gobies began experiencing stress due to increasing salinity at 21 ppt in the warm-water treatment. As a confirmation of stress at 21 ppt, individuals held at a sustained 21 ppt had significantly lower growth and HSI compared to those at ≤15 ppt, demonstrating this salinity as a potential threshold for long-term survival and establishment in warmer waters. In contrast, fish held at 5°C tolerated full-strength marine salinities (33 ppt) with no signs of increased stress response or increased mortality suggesting round goby may be able to overwinter throughout North American coastal estuaries and near-shore marine environments.

Our results shed light on the ability of round goby to temporarily tolerate salinity levels associated with lower portions of coastal estuarine systems and near-shore marine environments if environmental parameters are favorable. Furthermore, these findings demonstrate round gobies originating from a North American freshwater system have a higher salinity tolerance than previously documented. Karsiotis et al. [42] conducted similar step-wise experiments and found only 30% survival at 25 ppt in contrast to the results of this study exhibiting 100% (20°C) and 88% (26°C) survival at 24 ppt. The later onset of mortality in our study could be attributed to using more gradual salinity increases, allowing for longer acclimation periods, compared to the more rapid salinity increase schedules (i.e., 5 ppt every 3 days) used in Karsiotis et al. [42]. Longer acclimations periods used in our study may better reflect the potential natural expansion of round goby throughout major portions of the Hudson River Estuary if round goby remain at certain salinity levels for extended periods of time. On the contrary, shorter acclimation periods utilized by Karsiotis et al. [42] may be more representative of salinity fluctuations in the mouth of the Hudson River Estuary where salinity levels may shift up to ~±7 ppt with each tidal cycle [77]. Thus, the successful expansion and establishment of round goby may be dependent on in-river location and the magnitude and duration of salinity fluctuations.

This is the first study to examine the effect of temperature on salinity tolerance of round gobies, and we documented significantly earlier mortality in warm-water (26°C) and significantly lower mortality rates at cold temperatures (5°C) compared to preferred conditions (20°C). In addition to mortality events beginning at lower salinities for round gobies held at 26°C compared to 20°C, vitality scores also indicated stress occurring 3 ppt sooner in warmer waters. This is likely the result of the combined increased physiological demands created by warm-water conditions in conjunction with high salinity environments. Even without the additional osmoregulatory requirements, routine metabolic rate of round goby at warmer temperatures (24–28°C) can be 4–8 times higher than at cooler temperatures (0–5°C) [49–51]. Higher routine metabolic rates in fishes results in larger baseline energetic demands to maintain necessary oxygen levels and internal osmolality, but some fish may keep up with these increased demands if no other compounding environmental stressors (e.g., salinity) are present. Therefore, we can infer control fish in the warm-water treatment were able to keep up with their accelerated metabolism, as external signs of stress and mortality were absent. In addition to summer temperatures, a warming climate may hinder the southward expansion and establishment of round goby.

In contrast to warmer waters potentially inhibiting expansion into marine environments, we found that cold-water temperatures may facilitate survival in high saline waters. Round gobies in cold-water conditions (5°C) demonstrated behaviors consistent with overwintering fish (e.g., limited movement and lapse in feeding), likely resulting in the high survival within both experimental and control groups. Fish exhibiting little to no movement in winter conditions tend to maintain lower metabolic rates and decrease locomotor activity as a means to reduce energy demands and extend energy reserves [78]. Further research examining the effects of cold-water on gill morphology and osmo- and ionoregulation could provide insight into underlying cellular mechanisms (e.g., gill permeability, chloride cell composition, or Na^+^/K^+^ ATPase activity), allowing increased survival at high salinities. It is important to note that even though there was an increase in appetite in both experimental and control fish over the course of the nine-week trial, less than 30% of fish were eating. Our observations are consistent with reports that round goby undergo extended periods of minimal to no feeding in simulated winter conditions [49,79]. The sedentary period eventually ceases as results from Fortes Silva et al. [80] found that round gobies from invaded waters in Germany increased locomotor activity after 90 days at 5°C. Additionally, Glenn and Pennuto [81] noted round gobies captured in invaded Great Lakes tributaries were actively foraging in winter conditions (2–8°C) so it is plausible round goby from our study populations in invaded North American waters may continue to improve feeding and activity if held longer than the 75 days (acclimation + experiment) in our study. Further experiments are needed for understanding the adaptive capacity of round goby to not only survive long-term, but also to establish populations in colder waters with higher salinities in North American estuarine and even marine environments.

By examining the effect of temperature, reflective of seasonal conditions, on the salinity tolerance of round goby, we can provide insight into potential seasonal thermal refuge or offshore migratory patterns. The high salinity tolerance of round goby at low temperatures may be an important driver for further colonization of coastal environments. Round goby exhibit offshore migrations into deeper waters during winter months in the Great Lakes to overwinter in warmer and more stable waters [82–84]. If this migratory pattern occurs in an estuary, round goby could seasonally migrate through higher saline waters during winter months to expand into new habitats with more favorable conditions the following spring. For example, round goby may be able to temporarily survive the high saline waters in New York Harbor for long enough to gain access to Newark Bay, New Jersey and eventually the Passaic River, New Jersey where they could continue to expand upstream into waters with more favorable salinity levels. Round gobies could ostensibly capitalize on the apparent interaction of temperature and salinity tolerance to continue southward expansion through the Hudson River Estuary.

We postulate that early mortality in the cold-water control tanks could be attributed to time of collection of study specimens from the field. Round gobies were collected in June during spawning season [85] and all five fish that died were sexually mature, gravid females. Energy allocation prior to collection was most likely towards reproductive growth instead of energy storage which is required for overwintering [86]. Subsequently, these fish may have had poor energy reserves when transitioned to simulated winter conditions. We predict if gobies were caught post-reproduction (i.e., November), energy allocation and body composition might be more favorable to survive cold-water acclimation [87].

While round gobies collected from the northeastern invasion front in North America were found to have a higher salinity tolerance than previously recorded for fish sourced from freshwater, changes in vitality metrics in addition to minimal growth and poor body condition provide clear evidence of stress with increasing salinities. In 20°C and 26°C, individuals exhibited all three vitality stress metrics at 24 ppt and 21 ppt, respectively, and these generally occurred 3 ppt earlier than mortality events, indicating while temporary survival can occur at higher salinities (≥24 ppt), long-term viability in these conditions may not be feasible. For instance, more than 50% of experimental specimens in 20°C and 26°C were no longer eating at 24 ppt, suggesting stress-induced appetite suppression despite increasing osmoregulatory demands. Based on vitality scores, it is indicative 21–24 ppt is the upper threshold of salinity tolerance in warmer waters, however further evidence from the sustained salinity experiment suggests 21 ppt may be a more realistic upper long-term limit.

By holding round gobies at sustained salinity levels for an extended period of 9–10 weeks, we observed further evidence of osmoregulatory stress through somatic growth and HSI measurements, which refined our assessment of an upper salinity threshold. Even though there was 94% survival at 21 ppt for nine weeks, further examination of growth and HSI leads us to conclude long-term exposure to this salinity level is detrimental to adult round goby survival and potential establishment. The mean HSI of individuals held at 21 ppt was 2.75 which was significantly lower than all other salinity levels tested and is lower than documented HSI (3.2–6.5) of wild round gobies captured in the Baltic Sea and Danube River [71,88]. Therefore, we infer that food intake was not sufficient to maintain basic metabolic rates and osmoregulation, let alone allow for any somatic growth at 21 ppt. This is also supported by the fact that we found no significant difference in weight of specimens pre- and post-experiment at 21 ppt, such that energy appears to have strictly been used for maintenance requirements rather than anabolic processes. Looking at other salinity treatments, round gobies held at 1, 9, and 15 ppt were not in an energetic deficit as corroborated by a significant increase in weight in addition to high HSI values. Interestingly, results from an experiment conducted at 20°C with round gobies collected from invaded waters in the Baltic Sea (3 ppt) differed from our results as their findings showed growth at 30 ppt, although growth rates were significantly lower than other treatments (0.1, 7.5, and 15 ppt) [43]. Karsiotis et al. [42], documented a significant difference in weights after four months between 0, 10, and 15 ppt, with the highest gain occurring at 10 ppt; we noted no significant differences in post-experiment weight after 2+ months in our three treatments at 1, 9, and 15 ppt. Comparably, Hempel and Thiel [43] also noted no significant difference in weight gain over their 12-week experiment between 0.1, 7.5, and 15 ppt. As suggested by Hempel and Thiel [43], the geographic origin of study specimens may cause differing salinity tolerances, impacting long-term survival, growth, and body condition at sustained salinity levels. Round gobies are known to have high genotypic diversity and phenotypic plasticity which may lead to rapid evolutionary changes to aid in adapting and tolerating higher saline waters [55,88].

We provide novel insight into the significant effect of temperature on the salinity tolerance of round goby from a newly invaded North American coastal watershed. Based on our results, it is plausible there may be seasonally dependent spread throughout estuarine systems with expansion opportunities occurring in colder months and expansion barriers occurring in warmer months. We anticipate that adult round goby expansion into brackish portions (<21 ppt) of invaded coastal estuaries may be feasible year-round. However, round gobies may be able to temporarily endure higher salinities of up to 33 ppt as found in lower portions of estuaries and near-shore marine environments given ideal conditions at colder temperatures. It is essential to note that the salinity tolerances we observed for fish sourced from eastern New York waters were evaluated under controlled, optimal, laboratory conditions, thereby eliminating additional stressors wild round goby may face. Such stressors include environmental variability, salinity fluctuations through tidal cycles, food variability, and predation threats among others. Thus, salinity tolerance in situ may differ from results presented here. Further, the range expansion and establishment of round goby throughout North American coastal watersheds depends on a multitude of factors beyond adult growth and survival, including successful reproduction and the viability of early life stages in saline conditions. Future studies examining underlying physiological mechanisms occurring in round gobies under salt stress, prior to mortality, can provide further insight into long term survival in estuarine systems. Additional future studies aiming at assessing reproductive capabilities and other life stage survival across salinity levels will be important for informing expected establishment of round goby in brackish and marine habitats.

## Supporting information

S1 File Photo depicting tank set-up for salinity tolerance temperature trials. (TIF)

S2 File Photos depicting a healthy, control specimen (left) versus a stressed, experimental specimen (right). (TIF)

S3 File Mortality data. Data underlying Fig 3. Daily survival curves of experimental round gobies at three temperature treatments.
(XLSX)

S4 File Vitality score data. Data underlying Fig 4. Average proportion of round goby exhibiting non-stressed behavior (eating, light coloration, reacting) across salinity levels at three temperature treatments.
(XLSX)
