# Supplementary figures and images for: Salinity tolerance of Round Goby: Informing invasion potential in North American coastal watersheds
Source: PLoS One. 2025 Apr 25;20(4):e0316327. doi: 10.1371/journal.pone.0316327 (PMC12026935; doi:10.1371/journal.pone.0316327)

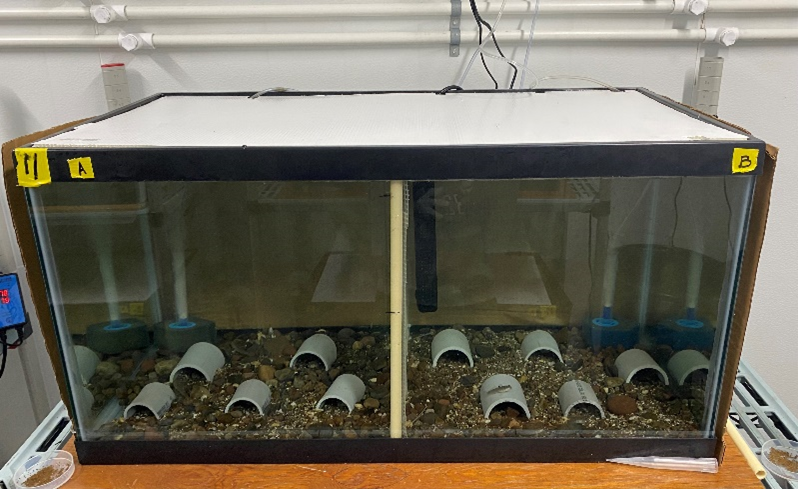

Supplement: S1 File — (TIF) [file pone.0316327.s001.tif]

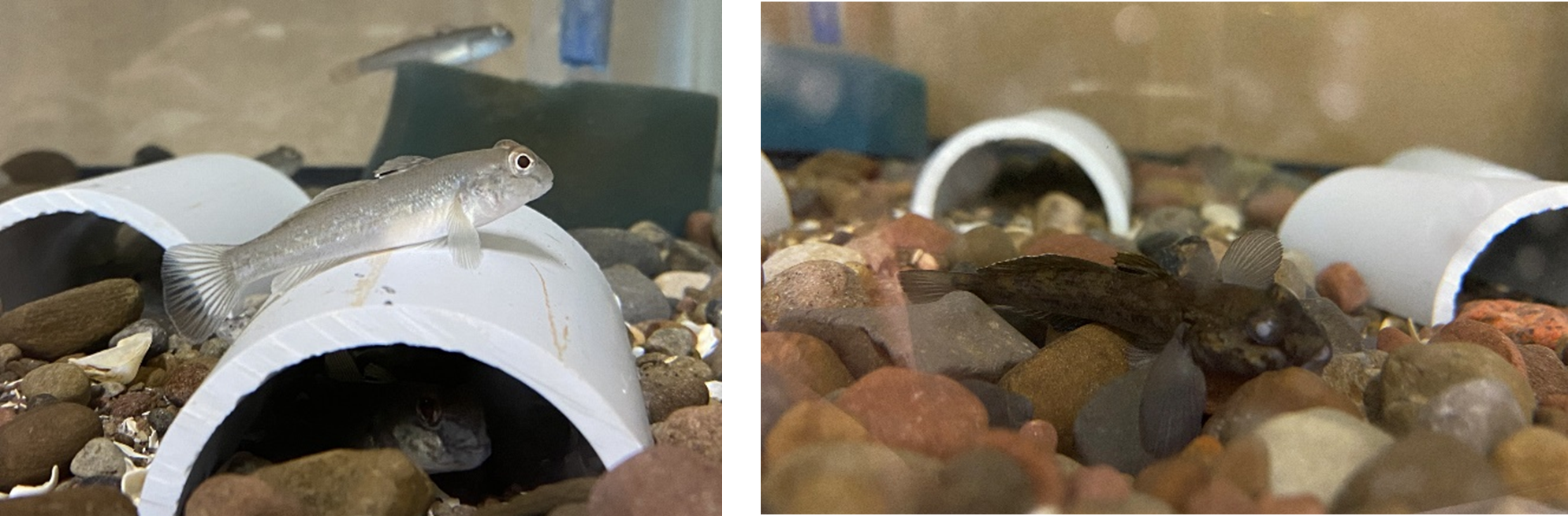

Supplement: S2 File — (TIF) [file pone.0316327.s002.tif]
